# Current approaches to secondary prevention after hip fracture in England and Wales — an analysis of trends between 2016 and 2020 using the National Hip Fracture Database (NHFD)

**DOI:** 10.1007/s11657-023-01282-2

**Published:** 2023-07-10

**Authors:** Zaineb Mohsin, M. Kassim Javaid, Antony Johansen

**Affiliations:** 1 Fracture Liaison Service Database (FLSDB) Clinical Fellow, Royal College of Physicians, NDORMS, University of Oxford, London, UK; 2 FLSDB Lead, Royal College of Physicians, NDORMS, University of Oxford, London, UK; 3 National Hip Fracture Database (NHFD) Clinical Lead, Royal College of Physicians, London, UK; 4 University Hospital of Wales and School of Medicine, Cardiff University, Cardiff, UK

**Keywords:** Osteoporosis, Hip fracture, Audit, Secondary fracture prevention, Hip fracture database

## Abstract

**Summary:**

Hip fractures are strong risk factors for further fractures. However, using the National Hip Fracture Database, we observed that in England and Wales, 64% of patients admitted on oral bisphosphonates were discharged on the same and injectable drug use varies from 0–67% and 0.2%-83.6% were deemed “inappropriate” for bone protection. This variability requires further investigation.

**Introduction:**

A key aim for the National Hip Fracture Database (NHFD) is to encourage secondary fracture prevention of the 75,000 patients who break their hip annually in the UK, through bone health assessment and appropriate provision of anti-osteoporosis medication (AOM). We set out to describe trends in anti-osteoporosis medication prescription and examine the types of oral and injectable AOMs being prescribed both before and after a hip fracture.

**Methods:**

We used data freely available from the NHFD www.nhfd.co.uk to analyse trends in oral and injectable AOM prescription across a quarter of a million patients presenting between 2016 and 2020, and more detailed information on the individual type of AOM prescribed for 63,705 patients from 171 hospitals in England and Wales who presented in 2020.

**Results:**

Most patients (88.3%) are not taking any AOM when they present with a hip fracture. Half of all patients (50.8%) were prescribed AOM treatment by the time of discharge, but the proportion deemed ‘inappropriate for AOM’ varied hugely (0.2–83.6%) in different hospitals. Nearly two-thirds (64.2%) of those previously taking an oral bisphosphonate were simply discharged on the same type of medication. The total number of patients discharged on oral medication fell by over a quarter in these five years. The number discharged on injectables increased by nearly three-quarters to 14.2% over the same period, but remains hugely variable across the country, with rates ranging from 0–67% across different units.

**Conclusion:**

A recent hip fracture is a strong risk factor for future fractures. The huge variability in approaches, and in particular the use of injectables, in different trauma units across England and Wales requires further investigation.

**Supplementary information:**

The online version contains supplementary material available at 10.1007/s11657-023-01282-2.

## Introduction

A hip fracture in an older patient occurring after a fall or trip, is an important moment to intervene to prevent subsequent fractures due to osteoporosis. Each year the national clinical audit records data on the care of more than 65,000 people with hip fractures, over 92% of all such patients across England and Wales [1]. Hip fractures represent one of the most serious fractures an individual can sustain with a mortality of 22% at one year, and hospital costs of £1.1 billion each year [2]. Patients who sustain one hip fracture have a much higher risk of sustaining another [3]. According to an Irish study, 1 in 11 hip fractures is a second hip fracture [4] with forty-six percent of second hip fractures occurring within the first 3 years following the index hip fracture [5].

Appropriate prescription of Anti-Osteoporosis Medications (AOMs) is therefore a priority to prevent a second hip fracture, or other fragility fractures. Trauma units should realise that missing this opportunity can be costly in terms of a patient’s future health, independence and care costs from future fractures.

National and international [6, 7] guidelines therefore make secondary fracture prevention a key requirement of basic hip fracture care. Registries such as the National Hip Fracture Database (NHFD) provide uniquely detailed data on the care and outcomes experienced by hip fracture patients and feedback from the audit has decreased mortality [8].

One of the key aims of the NHFD is encouragement of secondary fracture prevention through bone health assessment as a core component of hip fracture care [1]. Information about the provision of bone health assessment is one criterion for hospitals to receive the incentive payment of NHS England’s ‘Best Practice Tariff’ and since 2011 this has been recorded, along with whether patients were prescribed oral or injectable AOM on discharge.

In this report, we describe trends in secondary fracture prevention within the NHFD from 2016 – 2020 and compare the types of AOM patients were taking before and after a hip fracture in 2020.

## Methods

For every patient presenting with a hip fracture in England and Wales, the NHFD collects data to help inform local quality improvement, including data describing bone health assessment and secondary prevention following a hip fracture. In 2020, the latter was extended to include type of AOM on admission and discharge for each participating NHFD site, compared to documentation of oral versus injectable use previously.

Data entry into the NHFD is carried out by the clinician, health care professional or responsible administrative assistant. Clinical information regarding previous AOM use has been obtained in the process of history taking on admission from the patient, family or available information from online General Practice (GP) or Electronic Patient Records (EPR). We do not have information on why patients were prescribed an AOM prior to presentation with their index hip fracture but assume it is likely for primary or secondary prevention of fractures.

To minimise the burden of data collection, the NHFD does not require hospitals to record vitamin D and/or calcium; viewing appropriate supplementation as a prerequisite for more effective approaches on which its data collection is focused.

The NHFD makes such data freely available to local clinical teams and the general public on its website www.nhfd.co.uk, and we used such data in the analyses for this report.

## Results

NHFD annual reports have presented data from a quarter of a million patients over the age of 60 between January 2016 and December 2020.

Data of 63,705 patients from 171 hospitals were entered into the NHFD in 2020. Patients’ mean age was 83 years and the majority (70.5%) were women.

A comparison of bone management before and after the index hip fracture are shown in Table 1, with information expanded into individual oral and injectable medication in supplementary Table 1.

**Table 1:** Patients on Oral and Injectable Anti-Osteoporosis Medication on Presentation with a Hip Fracture vs. Medication Prescribed Post-fracture in 2020

Use of anti-osteoporosis medication at time of presentation with a hip fracture						
		Oral	Injectable	Not taking treatment	Missing	Total
Anti-osteoporosis medication prescribed after hip fracture	*n*	**6417**	**970**	**56274**	**44**	
	Oral	66%	2.50%	33.60%	11.40%	**67**
	Injectable	13.60%	82.40%	13.30%	0.00%	**23172**
	No assessment or action taken	0.20%	0%	0.40%	0%	**9165**
	Not needed/inap	2.50%	0.30%	7.50%	4.50%	**252**
		8.60%	6.80%	26.70%	9.10%	**4408**
		9.10%	8%	18.40%	2.30%	**15628**
		0.00%	0%	0%	72.70%	**11013**
		100%	100%	100%	100%	**63705**

Oral medication included: alfacalcidol/calcitrial, alendronate, zoledronate, ibandronate

Injectable medication included: zaledronate, denosumab, teriparatide

The majority of patients (88.3%) who presented with this index fracture were not on any anti-osteoporosis medication (AOM) prior to sustaining a hip fracture. Of the remaining patients who were on treatment for either primary or secondary prevention; 10.2% were on oral AOMs, 1.5% were on injectable AOMs and 0.1% had missing data.

The proportion of patients discharged on an oral AOM fell by over a quarter from 49.7% in 2016, to just 36.3% in 2020. Over the same period, the numbers started on injectable AOMs increased, so that, in total one in seven (14.3%) of all patients were discharged on an injectable AOM in 2020. The trends in secondary prevention therapy over a five-year period are summarised in Supplementary Table 2.

Of those patients who were not taking an AOM, (including the vitamin D analogues alfacalcidol and calcitriol) when they presented with the index hip fracture, about a third (29.4%) were prescribed an oral AOM by the time of discharge. A further 13.3% were started on an injectable AOM, of which zoledronate was prescribed twice as often as denosumab (9.0% vs. 4.3%).

Of the patients admitted with an index hip fracture who were already on an oral bisphosphonate (*n* = 4469 alendronate, *n* = 79 ibandronate, *n* = 548 risedronate) prior to admission, about two-thirds (i.e. 3231 of 5096) of the patients were discharged on the same class of medication (i.e. bisphosphonates) on discharge. One in seven (14.5%) were switched from an oral bisphosphonate to an injectable; denosumab and zoledronate being equally likely to be chosen as the new AOM (7.4% vs. 7.1%).

Overall, secondary prevention in the form of drug therapy (including activated forms of vitamin D) was offered to just over half (50.8%) of patients on discharge. Just over a third of patients (36.4%) were prescribed oral therapies and 14.3% an injectable.

Use of injectable medication varied from 0 to 67% in different units. Within parenteral AOM, the rates of denosumab (0.2%-63.80%), zoledronic acid (0.1%-64.40%) and teriparatide (0.1%-3.5%) also varied.

A quarter (24.5%, *n* = 15,628) were recorded as having been assessed but deemed inappropriate for bone protection, though many of these may have been started on calcium and/or vitamin D. However, the proportion of patients labelled in this way varied enormously (from 0.2% to 83.6%) in different hospitals, despite similarity between the patients cared for.

One in six patients (17.3%) of patients were discharged on no treatment pending bone densitometry and/or bone clinic follow-up and many of these will subsequently have been started on an AOM.

**Fig. 1:**
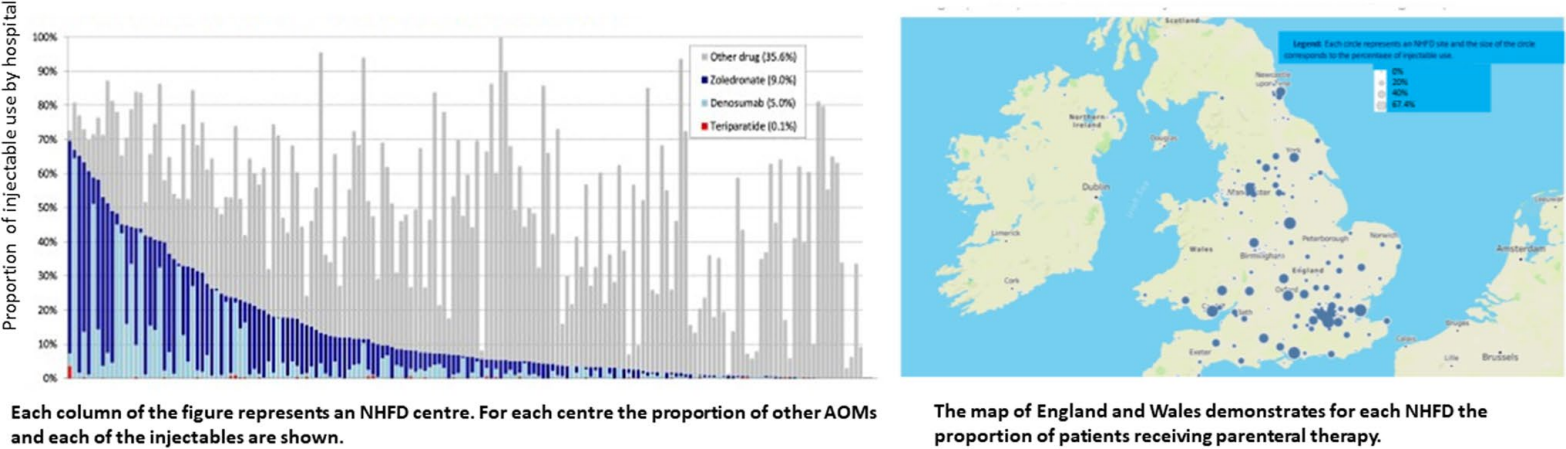
The graph shows the extent of variation in use of injectables between different hospitals and the map on the right shows how this variation is distributed across the country

Figure 1 shows the extent of variation in use of injectables between different hospitals, and how this variation is distributed across the country.

## Discussion

Our key findings were that 64% of patients admitted on an oral AOM were not switched to a more potent AOM on discharge and the marked increase in use of injectable AOM from 2016 to 2020 with one in seven patients now being discharged on such treatment with considerable variation between sites. This is despite a single NICE guideline for England and Wales and is likely more than can be explained by local differences in case-mix. Case-mix refers to the fact that comparison of hospitals must take account of differences in the mix of patients between providers by adjusting for known, measurable factors that are associated with performance such as age, sex, American Society of Anaesthesiologist (ASA) Grade, source of admission, mobility and fracture type [1].

Additionally, the percentage of patients for whom *no* assessment or action was taken doubled in 2020, likely indicating organisational constraints on orthogeriatric services, due to the COVID-19 pandemic [9].

A quarter of patients (24.5%, *n* = 15,401) were recorded as having been assessed but deemed not to require or be appropriate for bone protection. The NHFD’s annual report includes the actual rates of different AOM prescriptions for individual hospitals https://www.crownaudit.org/FFFAP/NHFD.nsf/docs/2021Report, and the huge variation in practice (from 0.2% to 83.6%) suggests that many of these patients are in hospitals which need to learn from their peers, and focus on this aspect of care in local quality improvement work.

Choosing the right AOM is a crucial as the phenomenon of an imminent fracture risk [10] following a fracture, whereby the markedly increased risk of sustaining a second fracture in the two years following an index fracture further heightens the urgency of managing these patients appropriately.

Assessment of bone health therapy in patients who present with a hip fracture should therefore include careful review of those who are already on treatment to evaluate treatment failure due to lack of adherence, treatment efficacy or severity of osteoporosis, and to consider more potent therapies in the high-risk patient [11–13]. In such patients, it is also important to check for secondary causes of osteoporosis including myeloma and coeliac screen, plasma parathyroid level, testosterone, prolactin, tests of cortisol excess and thyroid function tests as appropriate in line with international guidelines [14]. Attention to vitamin D replenishment is important, especially if planning parenteral treatment.

Studies have demonstrated that adherence to oral bisphosphonates is low [15]. Further, even in patient with high level of adherence, fractures on treatment were predicted by older age and dementia, highlighting patient groups requirement closer monitoring after recommendation of AOM [16].

Cognitive impairment (dementia and delirium) is common in this cohort of older patients, and adherence to oral therapy is challenging. It is therefore important to consider whether injectables might be the treatment of choice to ensure adherence (e.g., iv zoledronic acid) or to optimize treatment (e.g., denosumab). A post-hoc analysis of the HORIZON trial [17] showed that the beneficial effect of zoledronic acid appeared to be maintained for fracture reduction in those with cognitive impairment. This was despite patients with cognitive impairment having more risk factors for fractures and falls when compared to those with normal cognitive function.

As the use of injectables has become more acceptable with GP services able to administer denosumab injections in the community [18], a predictable increase in its use can be seen, with a corresponding fall in the initiation of oral AOM on discharge. Hospital level characteristics and patient level data may have influenced the frequency of injectable use in different units, and this can perhaps be the topic of a future QI project. Future work could also include following patients to see if the medication switch or choice on discharge has altered refracture rates within the next two years and if this is related to known areas of deprivation within the UK.

This study is the first data recorded by a national hip fracture registry detailing the AOM prescription on admission vs. discharge. The major strength of this study is the robust collection of data for the NHFD which has coverage of all NHS hospitals that treat hip fracture patients with 97% [1] having a bone assessment recorded.

Limitations pertain to registries in general and relate to incomplete, missing or incorrectly entered data [19]. In particular, the method of ascertaining AOM use at the time of the index fracture varied between centres.

In this report, we have endeavoured the describe the variation in injectable AOM use across different trauma units in England and Wales over time, despite the presence of a single national clinical guideline. We recognise that a limitation of this approach is considering all injectable AOMs in one category which includes medications that have a different mechanism of action, frequency of administration and cost. However, we hope this approach gives an overview of how local interpretation of national guidelines has led to considerable variability in type of AOM prescribed and the trends in use of injectable therapy over the past 5 years.

Another specific limitation of this study is the NHFD’s focus on treatment at the point of discharge after a hip fracture. The rates of AOM we have described will underestimate the eventual total number of people for whom an AOM was prescribed, since one in six patients (17.3%) were discharged on no treatment pending bone densitometry and/or bone clinic follow-up. Many of these will subsequently have been started on an AOM.

The NHFD does not currently record whether patients are still taking AOM at follow-up, though a new key performance indicator (KPI 7) will challenge hospitals to record the proportion of their patients known to still be on an AOM 120 days after their hip fracture [1].

## Conclusion

In this analysis of the NHFD, just over half of patients (50.8%) were on treatment at the time of discharge-either an injectable has been given, or an oral AOM has been started and included in the discharge medication list. A further 17.3% of patients may have received treatment after discharge following later review of the results of bone densitometry and/or follow-up in a specialist bone clinic. Most patients who were on an oral bisphosphonate on admission were discharged on an oral bisphosphonate without escalation of treatment. Approximately, a quarter were recorded as having been assessed but deemed inappropriate for bone protection, with a wide variation. The use of injectable AOM has increased by nearly three-quarters since 2016 but we have identified huge inter-hospital variation in practice. Cognitive impairment and co-morbidities will make oral therapies less reliable, and poor adherence and the severity of osteoporosis suggest that many hospitals need to learn from the proactive approach being taken by other teams dealing with the same frail, older high-risk population.

### Supplementary information

Below is the link to the electronic supplementary material.

Supplementary file1 (TIF 147 KB)

Supplementary file2 (TIF 136 KB)
