# Supplementary figures and images for: Current approaches to secondary prevention after hip fracture in England and Wales — an analysis of trends between 2016 and 2020 using the National Hip Fracture Database (NHFD)
Source: Arch Osteoporos. 2023 Jul 10;18(1):93. doi: 10.1007/s11657-023-01282-2 (PMC10333382; doi:10.1007/s11657-023-01282-2)

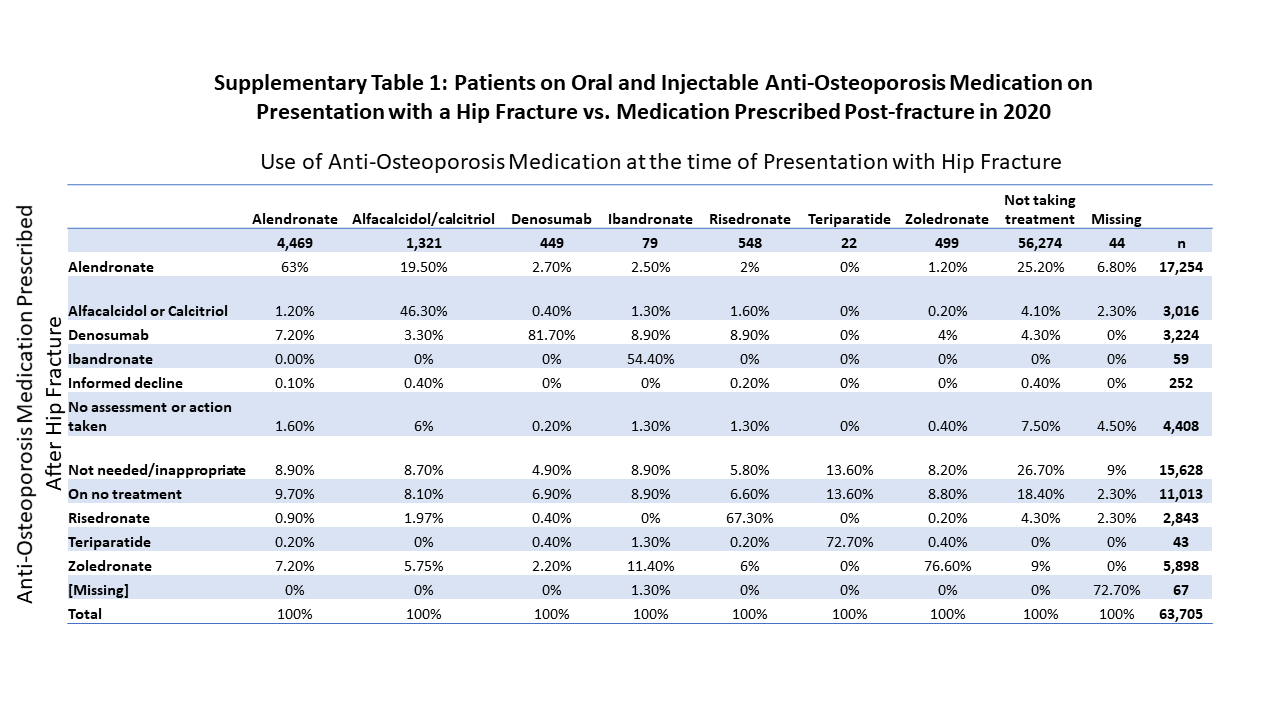

Supplement: Supplementary file 1 — Supplementary file1 (TIF 147 KB) [file 11657_2023_1282_MOESM1_ESM.tif]

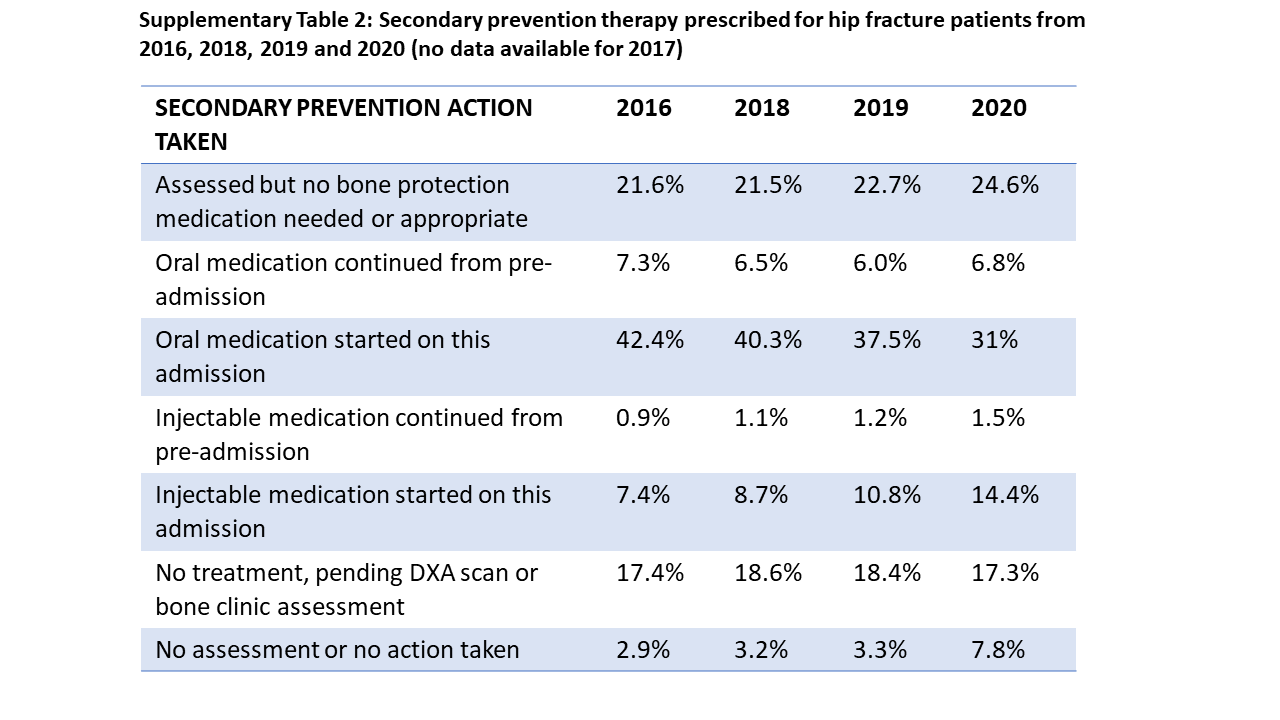

Supplement: Supplementary file 2 — Supplementary file2 (TIF 136 KB) [file 11657_2023_1282_MOESM2_ESM.tif]
